# Computing Workflows for Biologists: A Roadmap

**DOI:** 10.1371/journal.pbio.1002303

**Published:** 2015-11-24

**Authors:** Ashley Shade, Tracy K. Teal

**Affiliations:** 1 Department of Microbiology and Molecular Genetics, Michigan State University, East Lansing, Michigan, United States of America; 2 BEACON Center for the Study of Evolution in Action, Michigan State University, East Lansing, Michigan, United States of America; 3 Data Carpentry, datacarpentry.org

## Abstract

Extremely large datasets have become routine in biology. However, performing a computational analysis of a large dataset can be overwhelming, especially for novices. Here, we present a step-by-step guide to computing workflows with the biologist end-user in mind. Starting from a foundation of sound data management practices, we make specific recommendations on how to approach and perform computational analyses of large datasets, with a view to enabling sound, reproducible biological research.

## Abstract 2

With the biologist in mind, this Community Page presents a practical strategy for computing workflows that emphasizes reproducible research.

## Introduction

In today’s technology-driven era of biological discovery, many biologists generate extremely large datasets, including high-throughput sequencing, proteomic and metabolomic spectra, and high-content imaging screens. Subsequently, biologists must overcome many challenges of incorporating computing into their standard procedures for data analysis, including installing and running software that is not “point-and-click,” navigating at the command-line interface, comparing various analysis tools that supposedly perform the same tasks, establishing effective note-taking for their computing trials, and managing large datasets. Especially for trainees, it can be overwhelming to know how to begin to address these challenges. Providing a roadmap for workflow approach and management is likely to accelerate biologists’ skill-building in computing.

There are ongoing conversations regarding best practices for computing in biology. In 2009, arguably just prior to the “big data” deluge, Noble provided a great perspective on organizing bioinformatics projects [1]. Recent works have promoted methods for reproducibility in computational research [2,3], provided guidelines for writing software and tools [1,4–6] and advocated for training for the next generation of scientists in computing [7–9]. Aiming to complement and extend upon these works with the biologist end-user in mind, we outline an approach to computational analysis of any large dataset. The roadmap emphasizes tasks that help the user to understand the many choices that inevitably are made as part of a computing workflow. The intent is for the user to systematically query choices in software and parameters, compare options to understand the impact of each on the outcome and interpretation of results, and, ultimately, to make analysis decisions that are most appropriate and biologically or statistically defensible for the dataset in hand. Our roadmap also includes a series of specific internal (e.g., within a research group) and external (e.g., at peer review) checkpoints for reproducibility, provides suggestions for effective note-taking in computing, and advocates a team approach to sharing responsibility for analysis and data management.

It is important to emphasize that a good workflow starts with good data management and organization practices. The starting place for analyses is (1) primary (“raw”) data, the unprocessed data generated from a specific technology such as a sequencing machine, and (2) the data about that data, or *metadata* (Box 1), that contains information about how the data was collected and generated. Unmodified versions of both raw data and metadata should be stored securely, read-only (so it can’t accidentally be modified) with backups. If any manipulation has been done to the dataset from a facility for quality filtering or other processes, that information should also be retained as part of the metadata. Finally, initial data files should be organized so that they are computer-readable and in nonproprietary formats. These files are the cornerstone of any project and serve as a starting place for reproducing results, starting new analyses or continuing further work. These recommendations have been well outlined in [2,3] and are foundational for effective and reproducible analyses. This paper builds on that foundation, and focuses on the next steps: analysis workflow best practices.

**Box 1. Terms Used in This Paper**

- *approach*: a variable within a tool that specifies a statistical/bioinformatics process; e.g., an operational taxonomic unit (OTU) picking algorithm in amplicon analysis
- *control analysis*: using provided, simulated or mock data to ground expectations of software performance
- *metadata*: descriptors (may be qualitative or quantitative) of the biological (e.g., experimental conditions) and technical (e.g., sequencing protocol used) aspects of the data
- *parameter space*: all possible variables, methods, and tools available to analyze a dataset
- *parameterization*: the process of determining the “best” option for a given dataset or analysis problem
- *raw data*: the un-manipulated data exactly as it is returned by the technology that generated it
- *sanity checks*: small tests to ensure that script input and output files match expectations
- *sensitivity analysis*: the process of determining which options are most robust to outcomes in the analysis; sometimes called *sensitivity scan*
- *tool/software*: a compilation of functional code with or without a user interface that performs a statistical or bioinformatics task on a given input dataset
- *variable*: the user-defined options within a single tool. Default values are set by the developer(s); also called *flag* or *option*
- *version control*: automatic management of user edits to any type of document, including code and workflows. Typically records timestamp, author of the change, and the exact revisions to the file.
- *workflow*: the complete set of steps required to analyze a dataset from start to finish

## A Roadmap for the Computing Biologist

### i. Consider the Overarching Goals of the Analysis

Before beginning analyses, call to mind the goals of the project. For instance, working to address a given hypothesis will motivate different analysis strategies than conducting data exploration. A clear vision as to the ultimate project goals will direct the workflow, constrain analysis choices, and keep users on task to achieve their objectives.

### ii. Adopt an Iterative, Branching Pattern to Systematically Explore Options

Biologists embarking on the study of a new dataset face many analysis choices. Particularly in bioinformatics, at each step of the process there are multiple software available, each with its own set of variables. Because datasets have unique designs, biases, and underlying assumptions, a default option offered by a software is often not the most appropriate choice and must be re-evaluated for a new dataset. Biologists therefore often decide to try several software packages and analysis variables to determine the best match for their data type and set of questions. This set of all possible combinations of values for the different software and their options can be thought of as *parameter space*. Here we refer to *parameters* as the set of all possible options and *variables* as the options within a given software package. This is analogous to wet bench work, in which there are multiple steps or even protocols in each experiment and decisions must be made as to what components most affect experiment outcomes or are important to vary to optimize results (Table 1). Exploring this parameter space is an important step for a biologist to understand what the tool does and how it performs, and also to interpret the results for the specific dataset in question. However, it is challenging to keep track of the choices and results to effectively evaluate the most appropriate workflow (see section iv: “Taking Notes for Computational Analyses”). We suggest using an iterative, branching pattern for exploring parameter space, employing *sensitivity analysis*, *sanity checks*, and the potential use of *control analyses*.

**Table 1:** Analogies between computing and “wet-bench” experiments.

Computing task	Wet-bench analogy	Example
Exploring parameter space	Using multiple experimental designs to address a hypothesis	Complementing in vitro and in vivo (and in silico!) experiments
Comparing different computing tools or software	Comparing protocols that perform the same task	Comparing kits from different manufacturers for nucleic acid extraction
Changing variables (flags or options) within a computing tool	Making minor adjustments in a single protocol	Changing buffer conditions in a PCR

**Fig 1:**
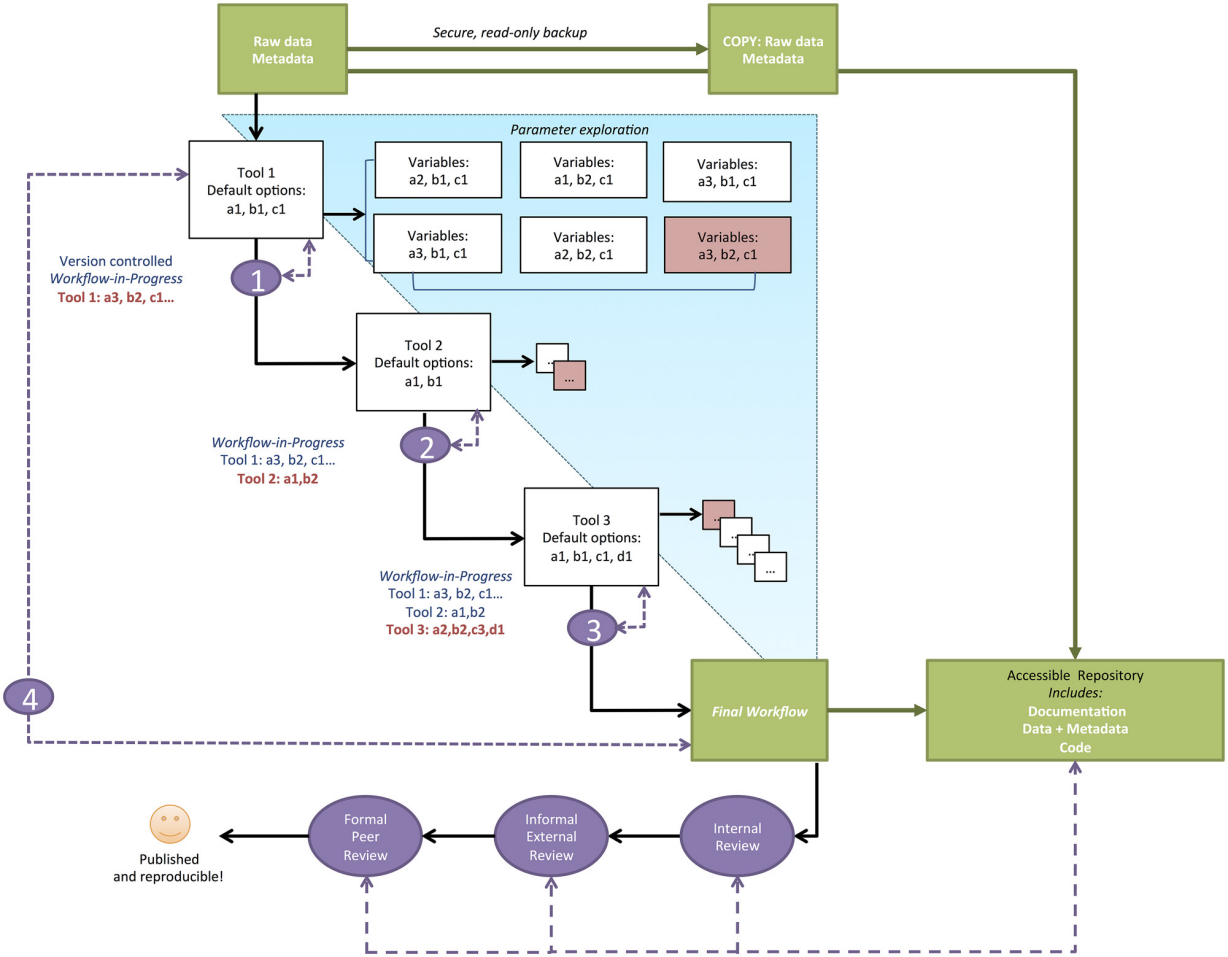
Workflow for biological computing. The workflow begins with read-only, secure raw data and ends with final code and data, ultimately accessible in a version-controlled repository (green boxes and arrows). Default and alternative parameters are explored and compared for each tool to optimize the analysis, and best choices (red boxes/text) are informed by biological and statistical expectations of the data. Purple ellipses show reproducibility checkpoints, with self-checkpoints numbered consecutively (here, 1 through 4). Purple dashed lines show iterative steps in the workflow that occur at reproducibility checkpoints. The workflow-in-progress is edited at every step until the documentation and code are finalized.

In *sensitivity analysis*, the researcher determines how the outcomes of the analysis are affected by each of the parameter choices (Fig 1). In this approach, one tool and its defaults are selected as a starting point. The user then changes the default value for each variable in turn and determines how each change affects the analysis outcomes. Values can range from defaults to those that are extreme or nonsensical to bracket expectations of potential outcomes. For every change in a variable imposed by the user, the corresponding input and outcome are documented. This process allows the biologist to determine which variables are most important for a given dataset, and while there may not be a universal “correct” value for each variable, the researcher can evaluate trade-offs and make an informed selection of appropriate values. When the final selection is made for each variable, she can record and annotate them with her rationale for the choices.

**Fig 2:**
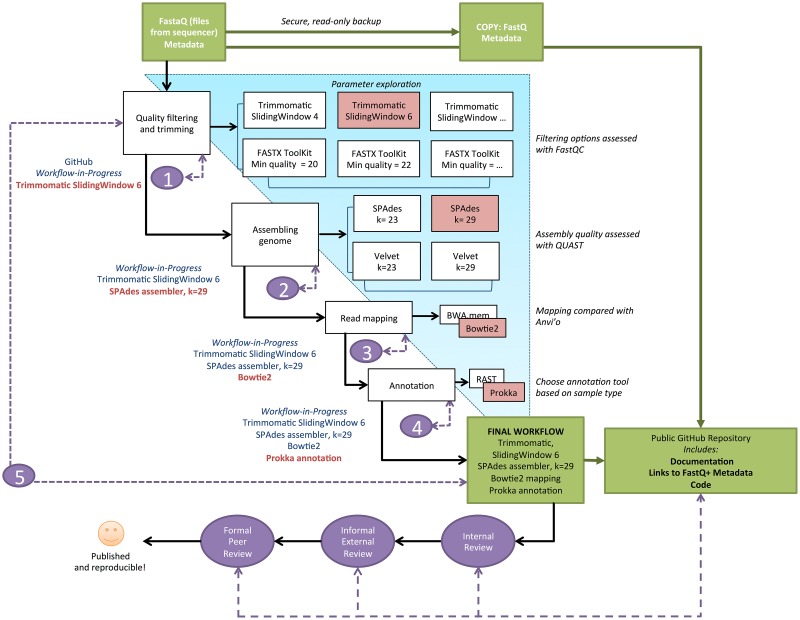
A simplified schematic of an example workflow for bacterial or archaeal genome assembly. These tools represent just a subset of those available, for illustration purposes. For example, Trimmomatic is one tool for trimming Illumina FASTQ data and removing adapters.

The biologist then moves on to the next step in the workflow or evaluates another tool at that same stage of the workflow. Exploration continues for each step independently, while the user refines a final workflow with the best variable values, methods, and tools at each step. Often, the user may want to return to a previous step to ask how changing a parameter influences a subsequent step. An example schematic for microbial genome assembly is provided in Fig 2. Notably, an independent software may be needed to evaluate analysis choices. For example, in Fig 2, FastQC is a software that can be used to assess and compare the effectiveness of different quality-filtering tools.

In light of the many alternatives at each step, it is important to remain focused on the ultimate task at hand, which is to address the hypothesis for which the dataset was generated. We do not recommend exhaustively exploring all parameter space, but to carefully consider those tools and variables that are important for the dataset and hypothesis. In the beginning of analysis, new users often require more time devoted to exploring options so that a greater understanding of the tool can be achieved. However, we caution users to not spiral down an analysis path of endless options. There are simply some options that do not matter for the outcome of the analysis, and these should be identified quickly and not considered further. For biologists just beginning in computing, making an in-person appointment with a bioinformatician, computing core facility staff member, or knowledgeable colleague can provide invaluable insights into which analysis options matter.

For new users, especially, self-imposed *sanity checks* are a helpful component of workflow development. These are small tests conducted while running software or executing code to make sure that the output matches expectations. For instance, does the number of sequences being returned match the number input or what was expected to be retained? Is the output returning integers or nonsensical values? Is the script to remove headers actually removing them and how could this be checked? Incorporating frequent sanity checks into the workflow will help the user to understand how the software and scripts are working and avoid “black-box” thinking, in which input and output are assumed to be correct. It also will strengthen the computing skills of the user as she develops creative ways to test whether the tools are running properly.

Often, within complex biological datasets, researchers do not know what the outcome of their data analysis should be, and there is not a clean, directly comparable “control.” Instead, biologists ask if a particular analysis meets their biological and statistical expectations of their data, and further question whether that expectation is met differently when using other comparable analysis approaches (e.g., [10]). In order to ground expectations, one approach is to use a mock or simulated dataset, in which the expected outcome is known. This is most analogous to a positive experimental control and can be thought of as *control analyses*. Mock datasets have been used in microbial marker gene analyses, in which, in parallel to sequencing a “wild” microbial community, a mock community of known isolates in known proportions is constructed and sequenced to calculate an error rate [11]. Another control exercise to ensure that the software is performing as expected is to use the software first with the developer’s provided dataset as a tutorial, ensuring reproducibility of their results.

### iii. Reproducibility Checkpoints

Reproducibility checkpoints are places in a workflow devoted to scrutinizing its integrity (Table 2). When scrutinizing, there are three main considerations. The first is that the workflow (or step in the workflow) can be seamlessly used (it doesn’t crash halfway or return error messages), and the second is that the outcomes are consistent and validated across multiple, identical iterations. In other words, the computational experiment must be replicated and found to be reproducible [2]. Finally, results should make biological sense; it is possible to generate reproducible computational results that are biologically meaningless.

**Table 2:** Reproducibility checkpoints during the development and refinement of a computational workflow.

Type	On what?	By whom?
Self	• Every parameterized step in a workflow • Final, complete workflow • Final batch script	• User(s) who develops the analysis workflow
Internal	• Final, complete workflow • Final batch script	• At least one colleague in the research group • Research group leader/principal investigator (PI)
External	• Final, complete workflow • Final batch script	• Crowdsourcing (e.g., GitHub/BitBucket/R community) • Informal review (ArchiveX, PeerJ PrePrint) • Reviewers and editor of a submitted manuscript

When users are working in teams to develop a workflow, checkpoints often come automatically as different users try to repeat and expand on each other’s work [6]. However, an independent user may have no formal mechanism of accountability from teammates. Thus, we recommend that, as a workflow is being developed, the user self-imposes reproducibility checkpoints at the end of each step. The workspace should be cleared, and the step run from scratch with the dedicated input files. If computational run times are too long to be practical, we recommend sub-setting the dataset and working with this manageable subset to develop the workflow. After the last step, the entire workflow may be similarly run from scratch with a clear workspace (Fig 1, see self-checkpoint 4). At this point, the user has completed her first draft of the workflow, and may ask for an internal review by a colleague. The colleague should try to reproduce the results at each step and run the workflow without error, and they should test a batch script that automatically performs each step to come to the final results file. The team leader or principle investigator (PI) should be able to execute the workflow and reproduce the results. We recommend that the PI review the workflow before any manuscript is drafted so that there is an opportunity to identify errors in the workflow and correct them before conclusions are drawn from the results.

Afterwards, the input files, annotated workflow, and batch script may be made available for external review. An especially rigorous set of reproducibility checkpoints will occur if the workflow is crowdsourced to developer communities for informal review, such as through GitHub, BitBucket, or R communities, or through pre-review mechanisms like ArXiv or PeerJ pre-prints. Also, we strongly recommend making all of the relevant analysis files (demo datasets, full datasets, metadata, documentation, and code) available to the editor and reviewers when a manuscript is submitted, which will promote replication of the computational experiment [2].

Visualizing data at reproducibility checkpoints can help to evaluate the performance of the analysis. Embedding data visualization into the analysis workflow will also promote generation of reproducible figures that can be used for publication. Creating code and documentation for generating figures is another important aspect of reproducible science.

### iv. Taking Notes for Computational Analyses

From the first moment of an undergraduate research experience, most biologists are trained on the importance of keeping meticulous and up-to-date laboratory notes for posterity, reproducibility of experimental conditions, and attribution of new ideas or techniques. We recommend that biologists approach note-taking for computational projects with the same integrity and accountability. Notes for computing can be embedded directly into most scripting language documents as comments, often designated with a hash (#). Just as for wet-lab notebooks, graduate students and post-doctoral trainees should provide all computing notes to their research team leader when the trainee moves on to her next position. In making recommendations for organizing bioinformatics projects, Noble in 2009 considered each computational “experiment” with the same weight as a wet-lab experiment [1], and we champion this perspective and his suggestions therein.

Analysis notes document the consecutive steps taken to explore parameter space for each choice. Like wet-lab experimental notes, the motivation for each step of parameter exploration should be provided, and, afterwards, the test results should be summarized. These notes should also document the rationales for the ultimately selected options. We reiterate the advice of Sandve [3] in taking these analysis notes, especially the need to record all versions of software and to copy lines of executed code verbatim. We also point readers towards Gentzkow et al. [12], who provide excellent advice for workflow documentation (as well as advice regarding other aspects of more advanced workflow, like automation and version control). We recommend maintaining and updating analysis notes in parallel with the draft of the final workflow. There are several options for organizing computational notes, including Jupyter notebooks, Galaxy, Arvados, and knitr for R. Carl Boettinger's online lab notebook (http://www.carlboettiger.info/lab-notebook.html) offers an excellent example of a well-annotated computational notebook, and there are numerous examples of reproducible academic publications (https://github.com/ipython/ipython/wiki/A-gallery-of-interesting-IPython-Notebooks#reproducible-academic-publications). Examples and indexes such as these provide the community with good reference points. While there are many excellent repositories for these resources, such as FigShare, Dryad, GitHub, BitBucket, and others, discoverability of these resources remains a challenge, and work could be done to develop catalogs of exemplars.

All workflows should be maintained using version control so that changes are automatically recorded and authorship to any changes can be attributed easily. Version control is a way of automatically tracking edits to a file, and some non-programming examples include using the “track changes” option in a word processing document, or an automatic backup image of a hard drive. In the literature, there is unwavering support for implementing version control [3,4,12,13]. The sooner biologists become comfortable with the mechanics of version control, the simpler workflow documentation and management will be.

### v. Shared Responsibility: The Team Approach to Reproducibility and Data Management

We posit that integrity in computational analysis of biological data is enhanced if there is a sense of shared responsibility for ensuring reproducible workflows. Research teams that work together to develop and debug code, perform internal reproducibility checkpoints for each other, and generally hold one another accountable for high-quality results likely will enjoy a low manuscript retraction rate, high level of confidence in their results, and strong sense of collaboration.

There are several strategies for cultivating a team approach to computational analysis. First, shared storage and workspace, such as on a cloud server or high performance computing cluster, can facilitate access to all group data. Raw files from large biological datasets can be maintained as read-only in a shared storage that can be accessed by all group members. In this way, users will know that the data are both safe and viewable by teammates, which should increase analysis accountability and prevent misconduct in data manipulation. Second, version-controlled repository hosting services, such as GitHub, GitLab, or BitBucket can promote teamwork by providing easy access to workflows. Research group leaders may create an “organization” for their group and allow team members access to shared repositories where they can sync, share, and track projects easily. GitHub, GitLab, and BitBucket also provide private repositories that can be shared with editors and reviewers of submitted manuscripts for external reproducibility checkpoints.

Research group leaders can instill a sense of shared responsibility within their groups by setting expectations for frequent reproducibility checkpoints. For instance, PIs may ask trainees to demo workflows-in-progress during meetings. Another strategy that has been productive in our own experience is to host routine team “hackathons”: open-computer group meetings dedicated to analysis, including facilitating reproducibility checkpoints, sharing expertise and strategies, and collectively solving analysis hurdles. The hackathon format brings both experienced and novice coders together, creating a shared learning experience that can be productive and motivating [14]. Research group hackathons are also excellent venues for collective exploration of parameter space. For example, if each team member explores one option (in a single tool), a team of five can collectively compare their results and complete parameter optimization for that tool in minimal time as compared to the time an individual user would require. Routine group hackathons also keep the team leader informed of and accountable for the analyses performed by their group members. Finally, routine research group hackathons can promote internal reproducibility checkpoints resulting in incremental workflow revisions, which are suggested to be effective in improving code and finding errors [15].

Journal reviewers and editors are also a crucial part of the shared responsibility equation. They may request that code and raw data are available for review, and then also make earnest attempts to test-drive the provided workflows to ensure their integrity. For deviations in standard methods or new workflows, reviewers should not be satisfied with a batch workflow script. It is in the journals’ and the authors’ best interests to have the “guts” of the workflow examined. Reviewers should also be forthcoming with editors if they are unable to comment on the inner workings of the author-provided workflow so that editors can seek out additional reviewers that are able to provide appropriate critique. Editors may want to ask directly (for example, using a check box on the review form) whether reviewers attempted to reproduce the results or run the analysis code.

Not all biologists have immediate colleagues who can support their computing efforts. Many trainees may be the only person computing in their research group, which can be an isolating experience involving much trial and error. For these researchers, we suggest seeking out an intellectual community that can offer aspects of the team approach. Options include attending open office hours at the bioinformatics core or joining meetings of a computing research group on campus. It is also possible to query or ask questions on online help forums like BioStars, SEQanswers, or StackOverflow; interact with active community members on Twitter or on mail lists such as R-help or Bioconductor; and follow or comment on online blogs and articles. These activities allow biologists to engage with a virtual community of researchers and network with programmer colleagues. Tips for interacting in these forums include the following: (1) asking the proper question with as much clarity and detail as possible; (2) before asking a question, make an honest effort to troubleshoot by conducting verbatim queries of error messages with search engines and investigating the forums for similar issues that have been previously addressed; and (3) share code and provide a reproducible subset of the data for others to consider as they help to answer the question. It’s easier for others to understand and help if they have an example that recreates the error.

## Conclusions

Skills in computing can enhance biologists’ logic and capacity for experimental design, increase understanding and interpretation of results, and promote interdisciplinary science by building a shared vocabulary and experience with collaborators in computer science, bioinformatics, statistics, physics, and engineering. We’ve suggested a systematic roadmap for computing workflows for biologists, including considering the overarching goals of the workflow, taking an iterative approach to analysis, implementing reproducibility checkpoints, recording effective computing notes, and adopting a team approach to analysis.

Ultimately, it is the responsibility of team leaders to implement policies and establish expectations for large data analysis within their groups. Large data analysis in biology requires an incredible skill set that takes time and commitment for a user to develop. Group leaders can encourage trainees to spend the productive time needed to master a workflow, and also to carefully perform the analysis and interpretation. The more biologists trained to master basic computing, the more skilled our workforce for meeting tomorrow’s challenges in biological discovery, which, inevitably, will involve even larger datasets than we have today.
